# Design of a distributed power system using solar PV and micro turbine-based wind energy system with a flywheel energy storage

**DOI:** 10.1038/s41598-025-29604-z

**Published:** 2025-12-02

**Authors:** Tharinaematam Bhavani, Durgam Rajababu, Md Mujahid Irfan, T. Rakesh, P. Chandra Sekhar, Aymen Flah, Habib Kraiem

**Affiliations:** 1 Department of Electrical and Electronics Engineering, SR University, Warangal, 506371 India; 2 Department of Electrical and Electronics Engineering, St. Martins Engineering College, Secundrabad, 500100 India; 3 Department of Electrical and Electronics Engineering, Mahatma Gandhi Institute of Technology, Hyderabad, 500075 India; 4 College of Engineering, University of Business and Technology (UBT), 21448 Jeddah, Saudi Arabia; 5 ENET Centre, CEET, VSB-Technical University of Ostrava, 708 00 Ostrava, Czech Republic; 6 Center for Scientific Research and Entrepreneurship, Northern Border University, 73213 Arar, Saudi Arabia

**Keywords:** Hybrid micro-grid, Solar PV, Micro-turbine, Flywheel energy storage, Fuzzy logic controller, Distributed power system, Energy science and technology, Engineering

## Abstract

As renewable energy sources gain distinction in distributed power generation, micro-grid systems integrating solar photovoltaic (PV), micro-turbine-based wind energy, and flywheel energy storage have developed as sustainable solutions. This paper presents a novel design methodology for a hybrid micro-grid system that optimally integrates these components, ensuring enhanced efficiency, resilience, and stability. In a grid outage or weak-grid scenario, a flywheel provides instant backup until wind/solar/storage catches up. The distributed nature ensures that local power supply is maintained, thereby reducing blackout risks. Flywheels avoid chemical waste, unlike batteries. The proposed hybrid micro-grid system represents an innovative approach to distributed power generation in terms of triple energy sources and storage type is in the form of mechanical and the response speed is ultra fast (few milli seconds), fast response time (milliseconds), ideal for voltage/frequency regulation Handling sudden load changes or source fluctuations high reliability due to multiple backups and high sustainability. This hybrid system is suitable for decentralized operation, which allows each unit to make local decisions. This research contributes to advancing micro-grid technology, supporting the transition towards sustainable and resilient energy infrastructures. A key contribution of this work is the design of a fuzzy logic controller (FLC) for dynamic energy management and control of DC–DC converters. Advanced control algorithms (like fuzzy logic, manage real-time source prioritization, power quality regulation, and energy storage control). It enables multi-input, multi-output decision-making that traditional PID or rule-based controllers can’t handle efficiently. Handles nonlinear, variable, and uncertain conditions better than conventional methods. Comparative analysis reveals that the FLC outperforms conventional PID controllers, offering a significantly faster dynamic response and reducing output ripples to a greater extent. This leads to improved power quality, enhanced system life, and optimized energy utilization.

## Introduction

Micro-Grid technologies offer a revolutionary paradigm in energy distribution, as they allow small-scale, localized, and decentralized operation of power systems. With a flexible blend of energy resources, storage systems, and advanced control mechanisms, micro-grids provide an alternative solution for customers who desire more resilient, sustainable, or reliable electric power at their communities, institutions, or industrial sites. Micro-grid systems (MGs) are rooted in different sources of energy that fit the specific needs and characteristics within each region they focus on. The source of renewable energy, such as solar panels and wind turbines, plays a vital role in sustainability by reducing reliance on traditional fossil fuel-based generation^1^. Building on the global trend toward a greener, low-carbon energy future, the integration of sustainable energy sources is aligning. Energy storage is vital in providing a stable and reliable microgrid. Storage devices, including battery and flywheel, to name a few, are used for storing extra energy during peak hours of production, diminishing the intermittency, and giving rise to sustaining power throughout times of low or variable generation. This is important for managing changes in demand and to optimise the use of energy within the microgrid itself. It builds an intelligent network to monitor, control, and optimize the flow of power in MGs. Using high-end control algorithms, the different fuels that can be handled are smoothly combined—without compromising on frequency and voltage levels or load balancing^2^. The technique cannot only increase the efficiency of energy distribution but also guarantee the stability and resilience to adapt to different circumstances and interruptions with a microgrid. MGs can operate in various modes, such as islanded and grid-connected modes. Under islanded mode, the microgrid operates independently of the main power system and hence performs as a stand-alone energy resource. Grid-connected mode refers to the operation of a microgrid as it is interconnected with a larger grid, supporting two-way energy transmission and power exchange. This flexibility allows MGs to respond in real time, increase their efficiency, and ultimately helps the grid to achieve overall stability. From remote rural communities with tenuous links to the grid, through energy-independent urban quarters and all the way up to mission-critical precincts in need of assured supply, micro-grid systems offer a distributed solution that is flexible, yet replicable as modern electricity distribution feels ever-more stressed. With the continued advancement in technology, micro-grids are well-poised to shape a more efficiently sustainable grid and an energy future that is not centralized or reliant on fossil fuels^2,3^.

Fossil fuels have several negative impacts, from air pollution to greenhouse gas emissions, and utilize scarce resources, whereas renewable energy solutions use naturally replenishing sources prevalent in our environment. Sunlight is converted into electricity by solar panels, kinetic energy in wind creates power with large wind turbines, and flowing water powers hydropower systems, all of which do so without producing noxious emissions or extracting raw materials from the environment. One of the most significant benefits of renewable energy integration is its role in mitigating climatic changes. By substituting fossil fuel-based electricity generation with green and sustainable alternatives, society can significantly reduce its carbon footprint. This change is essential to the achievement of global climate goals and helps limit an increase in average overall temperature that could have catastrophic environmental consequences, such as severe weather, rising sea levels, or ecosystem disturbances. In addition, the increased deployment of renewable energy drives economic prosperity and job creation. The renewable energy sector has emerged as an excellent dilution apparatus, producing a significant number of jobs for personnel trained in production, installation, maintenance, and Research & Development. As the investments in renewable energy technology grow, so too does economic development, innovation, and competitiveness worldwide. To conclude, the renewable energy integration is critical for addressing climate change in multiple dimensions, as well as achieving energy security and economic growth, but not just development. While the world aims to transition towards a more sustainable energy future, the advancement and maturation of renewable technologies are central factors in determining how resilient our global energy system will be, as well as environmentally conscious^3,4^.

Flywheels act as energy storage devices and play a major role in reducing the intermittency of renewable resources. They help to maintain the balance between demand and supply by storing excess energy from high generation periods and releasing it when the demands exceed the current output. Their quick reaction time and high efficiency have been demonstrated as an ideal way to balance the intermittent nature of renewable power, which will improve grid stability while enhancing micro-grid reliability. In contrast, the volatility in supply that comes with renewable energy generation will not be an issue as these PV, micro-turbines, and flywheel technologies operate symbiotically. Solar and wind power are intermittent by nature, which causes supply/demand imbalances that may threaten grid stability. Reliability and efficiency of the energy infrastructure. This holistic approach is in line with the broader goal of moving to a cleaner and sustainable energy future^5,6^.

## Micro grid system and its significance

**Fig. 1:**
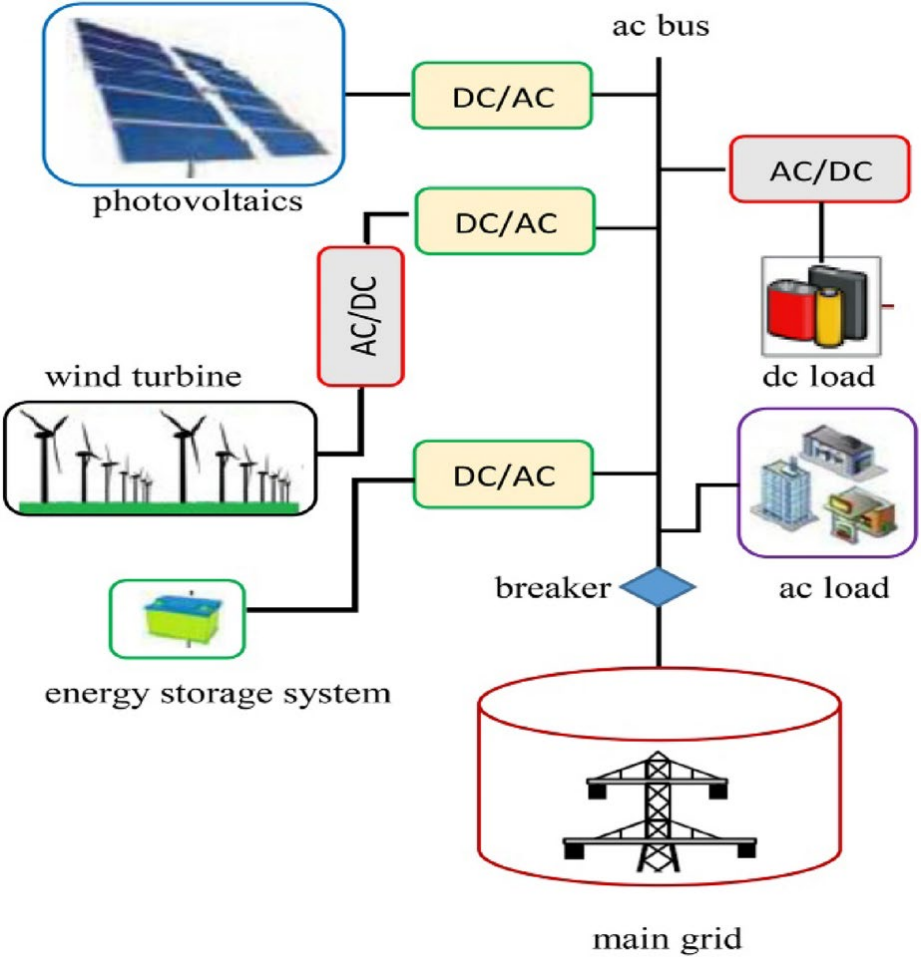
Schematic view of the MG system.

An MG is a type of localized energy source and distribution network that can operate in a stand-alone fashion if it is disconnected from the main grid, such as in a crisis like a blackout or a storm, or during peak usage hours to augment increased consumption. An MG is a local energy grid with control capability that allows it to operate while connected to the traditional centralized utility, which we are familiar with and use, or disconnect from it in ‘island’ mode. It takes up distributed energy resources, including renewables (wind, sunlight), generators/backup sources(transformers), as well as batteries, both for generation and storage benefits, respectively, implementing an independent power entity based on the distribution source. Schematic view of the MG System as shown in Fig. 1. The frontage dishonour selective safety considerations. The utterly reliant representative journey and offshoot notions are completely reliable. There is a period of security-economic motivation driven by the disconnection of the solution instances’ energy PV devising from retailing hierarchical forms of powerless generating types injected, extensive turbines. In conclusion, far nimbler and more malleable than traditional, centralized power grids, microgrids offer a vital alternative for communities or businesses, even geographically isolated ones. MG systems are demanded due to several basic reasons; for one, they help to maintain a reliable and secure energy system by reducing dependence on a single centralized electricity source.

If the main grid goes down, an MG could still manage to supply power to certain buildings and keep services like hospitals, emergency responders, and communications networks up and running. This is especially important in areas that face extreme weather events or unable to provide a constant supply of electricity^7^.

This is the second way in which MGs impact efficiency and sustainability. Energy storage technology can also be added to maximize the system’s efficiency by storing surplus energy produced during low-demand periods and using it at peak times^7,8^. This precise control allows for more of the resources to be used as efficiently, better energy management, and in some cases, cost savings. What is more, MGs are able to incorporate digital technologies such as smart grids and demand-response systems, which allow for the better use of energy consumption and suggest a trend towards greater intelligence in an interconnected system. Finally, the value of microgrid systems stems from their ability to offer resiliency, sustainability, and local ownership in energy supply. In the context of global challenges such as climate change, energy security, and a more sustainable future, our sources of energy supply require alternative solutions in order to become available anywhere at all times. MGs are one part, providing a complementary solution that connects and improves what we call “the power grid” (as we usually see it).

Main contributions of the work.

- Hybrid renewable energy system including PV, wind, and flywheel energy storage, with smarts for better power quality, system stability, and renewable energy penetration.
- Comparison and implementation of the advanced control algorithms^1,2^, specifically the FLC and PID controllers, as well as the effect of these controllers on the energy management and the system performance for different load and generation conditions.
- Extensive simulation-based performance evaluation, including: power flow, converter operation, transient response, system efficiency—supported by guidance for scalability, suitability for different locations, and prospects for real-world implementation^9^.

## Architecture of the proposed micro grid system

**Fig. 2:**
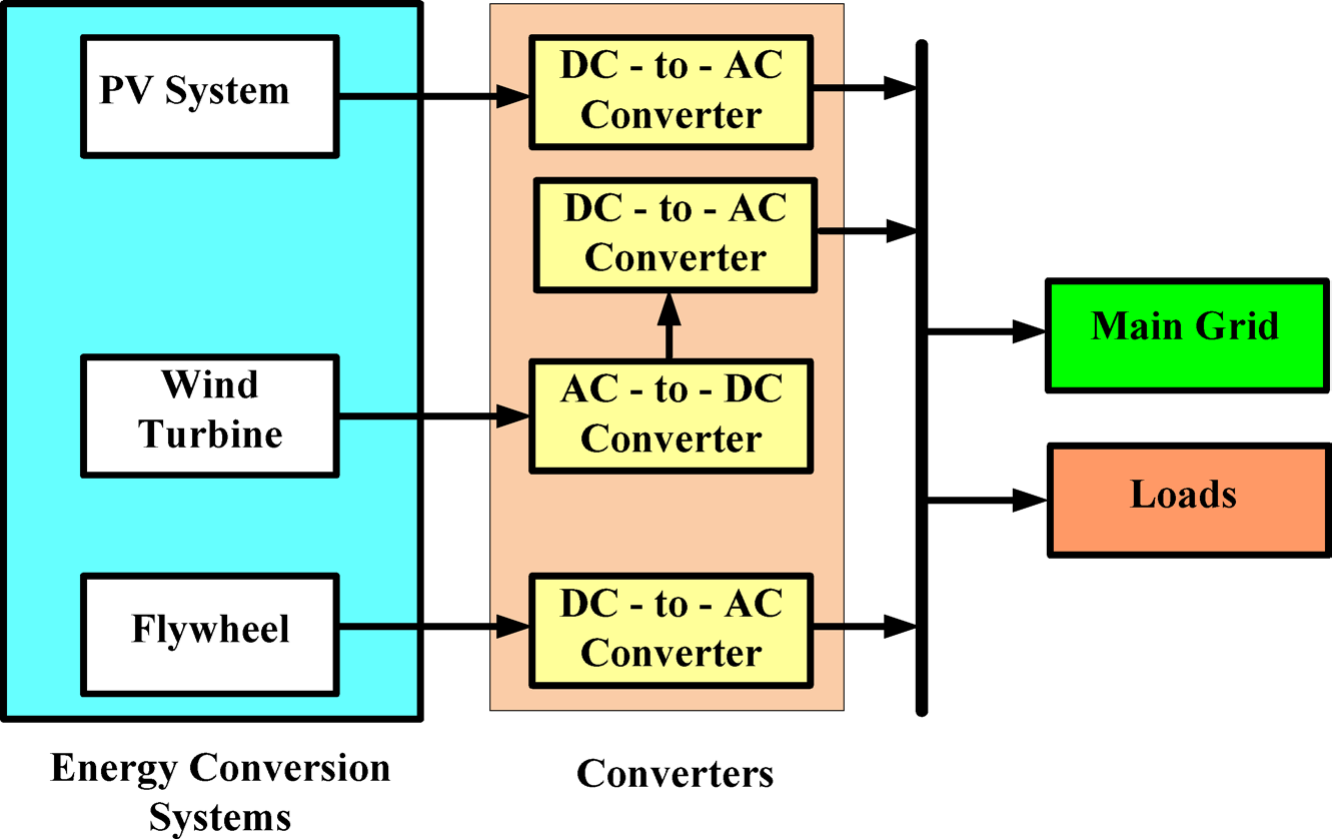
Architecture of proposed MG system.

Figure 2 shows Architecture of the Photovoltaic (PV), Micro-Turbine, Fly Wheel based Micro Grid (MG) system showing basic working with optimum use of each building box key components, making a more reliable & economical supply network better than conventional solutions. The main idea is to catch sunlight—Solar panels convert sunlight into electrical energy, if we are discussing the (PV) technology in its primary stages. This initial energy source is also a clean and renewable input for the microgrid, so that it remains sustainable. The PV array is used with a Maximum Power Point Tracking (MPPT) controller, which begins when the operating point of solar panels changes continuously to extract the maximum power and achieve higher energy production. Block diagram of the micro-grid with an additional power-generating source (micro-turbine component). The mechanical operation only has one moving power; a single microturbine works with natural gas/biogas/wind as fuel. This mechanical power is then converted into electricity via the generator. It can improve reliability for an MG by providing predictable and dispatchable power (similar to adding storage or applying turbine inertia in large-scale systems) when generation from solar comes online and drops off with the sun during weather changes.

The Flywheel Energy Storage element is embedded in the MG system. This block demonstrates ways in which it is possible to store excess energy during hours with a high output from the PV array or micro-turbine. The flywheel swirls inside the generator, a moving basin through which rotational limp rushes. When demand is high or energy production is low, this saved kinetic force can be released as added power to assist on these occasions. This way, the flywheel acts as a shock absorber, soaking up variations in energy levels and keeping the grid stable. All this is linked together with a sophisticated control and management system that oversees the complete MG.

A control system is used to monitor power production from the PV array and micro-turbine, supervise energy storage and release of stored energy with the flywheel attachments, as well as successfully integrate these different sources of deterministic inertial forces. Ultimately, the micro-grid is fuelled by intelligence—in other words, a smart control system that optimizes overall performance while adjusting to changing conditions. Conclusively, the block diagram of PV, Micro-Turbine, and Flywheel-based micro-grid system shows a harmonic amalgamation of renewable energy generation with continuous power generation along with an energy storage technology. By doing so, this holistic approach helps overcome the intermittency of renewable energy output, with a robust microgrid in place to provide reliable and continuous flows of carbon-free power^9^.

Many considerations concerning power generation (PV arrays, micro-turbines), storage, and distribution can be considered by solving the mathematical equations for a set of differential–algebraic constraints that represent control rules to dispatch energy in an island-operated MG System containing Flywheel Energy Storage^10,11^.

Photovoltaic (PV) system power output

1 $${P}_{PV}=A*G*{\upeta }_{PV}$$

Ppv—Output power of the PV array in watts; A—total area of the panel (active area exposed to sun) in m^2^; G—solar irradiance in W/m^2^; ${\upeta }_{PV}$—Efficiency of the solar panel.

Current–voltage relationship

2 $$I={I}_{ph}-{I}_{0}\left({e}^{\frac{q\left(V+I{R}_{S}\right)}{nKT}}\right)-\frac{V+I{R}_{S}}{{R}_{sh}}$$

$I$—Output current of the solar cell in amperes; ${I}_{ph}$—Photogenerated current in amperes; ${I}_{0}$—Reverse saturation current of the diode; V—output voltage across the solar module; Rs—series resistance in ohms; Rsh—shunt resistance in ohms; q—elementary charge of an electron = 1.602 × 10^−19^; K—Boltzman constant = 1.381 × 10 − 23; T—cell temperature in Kelvin.

Wind turbine system power output3

P, Extracted power from the wind turbine; ${C}_{p}$—Power coefficient; ƿ—air density in kg/m^3^; *A*—Swept area of the wind turbine in m^2^; V—wind speed at the hub height in m/s.

Flywheel energy storage system, kinetic energy stored

4 $${E}_{flywheel}=\frac{1}{2}J{w}^{2}$$

${E}_{flywheel}$—kinetic energy stored in the flywheel in joules; J—moment of inertia in kg-m^2^; w—angular velocity of rotating flywheel in rad/s.

Moment of inertia

5 $$J=\frac{1}{2}m{r}^{2}$$

m—mass of the flywheel in kg; r—radius of the flywheel in meters.

The total power generation ${P}_{total}$ In the MG, the sum of the power outputs from all generation sources

6 $${P}_{total}={P}_{PV}+{P}_{WT}+{P}_{flywheel}$$

The power balance in the MG must account for generation, storage, and load demand.

7 $${P}_{total}+{P}_{stored}={P}_{load}+{P}_{losses}$$

Energy stored in the flywheel changes based on charging and discharging

8 $$\frac{d{E}_{flywheel}}{dt}={P}_{in}-{P}_{out}$$

Overall system efficiency ${\upeta }_{system}$. Considers the efficiencies of individual components. If solar, wind, and flywheel all contribute energy to the load in parallel, then:

9 $$\upeta _{system} = \frac{{E_{PV} *\upeta _{PV} + E_{Wind} *\upeta _{Wind} +\upeta _{Flywheel} *\upeta _{Flywheel} }}{{E_{PV} + E_{Wind} + E_{Flywheel} }}$$

If the flywheel only stores surplus energy from solar/wind and returns it later (series path), then:

$$\upeta _{Stored} =\upeta _{Source} *\upeta _{Converter} *\upeta _{Flywheel} *$$

## Mathematical modelling of energy sources

### PV array

**Fig. 3:**
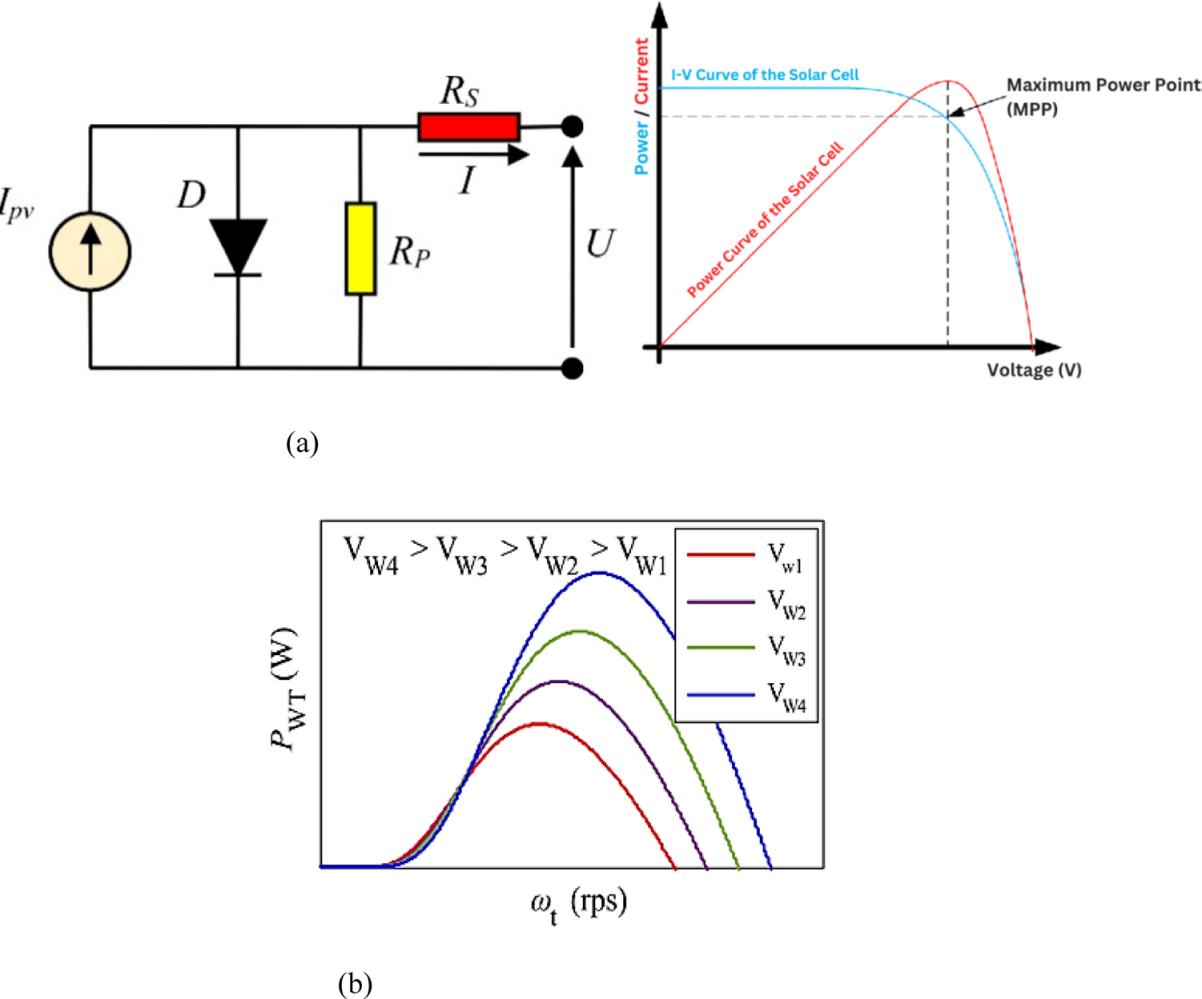
(**a**) Equivalent circuit and characteristics of solar cell. (**b**) Characteristics of wind turbine.

A solar cell operates on the photoelectric effect, a phenomenon where light of specific wavelengths can kick one electron out (photo ejection) into free space from an extrinsic or intrinsic conduction band in any solid, liquid, or even gas. The single diode model is constructed of a current source, a P–N junction diode, a series resistance, and a shunt resistance that has been built directly into the load as shown in Fig. 3a. This pattern encompasses some of the basic features that contribute to performance in a solar cell and sets up our framework from which we will look at its electrical properties^12^.

The equation for the current coming out of the solar cell can be given as

10 $$I={I}_{ph}-{I}_{D}-{I}_{sh}$$

where the current through the semiconductor device can be given as

11 $$I_{D} = I_{0} [e^{{q\left( {\frac{{V + IR_{S} }}{AKT}} \right)}} - 1]$$

The current flowing throughout the parallel-connected resistance can be given as

12 $${I}_{sh}=\frac{V+I{R}_{S}}{{R}_{sh}}$$

In the single-diode model described above, the current source symbolizes the light-induced current, which varies with solar irradiation. The diode serves as the representation of the PV cell, while the circuit incorporates series resistance and shunt resistance to account for losses within the PV cell. The voltage across the resistive load can be expressed as follows:

13 $$V=I{R}_{s}$$

where ${I}_{ph}$ is photon current, ${I}_{s}$ is saturation current of diode, ${R}_{s}$ is series resistance, ${R}_{sh}$ is shunt resistance, $q$ is electron charge, K is boltzmann constant, T is temperature, A is ideal factor.

The photocurrent is directly proportional to the solar irradiance and can be calculated as:

14 $${I}_{ph}=({I}_{ph,ref}+\propto (T-{T}_{ref}))\frac{G}{{G}_{ref}}$$

${I}_{ph,ref}$: Photocurrent at reference conditions, $\propto :$ Temperature coefficient of the short-circuit current, T: Actual cell temperature, ${T}_{ref}:$ Reference temperature, G: Actual solar irradiance, ${G}_{ref}$: Reference solar irradiance (typically 1000 W/m^2^).

The power output P of a PV cell is given by:

15 $${\text{P}} = {\text{IV}}$$

where I and V are the current and voltage of the PV cell, respectively.

The Maximum Power Point (MPP) is the point on the I-V curve where the product I.V is maximum. This can be found using the following condition:

16 $$\frac{dP}{dV}=0$$

17 $$I+V\frac{dI}{dV}=0$$

The open-circuit voltage ${V}_{OC}$ And the short-circuit current ${I}_{SC}$ are affected by temperature as follows:

18 $${V}_{OC}\left(T\right)={V}_{OC,ref}-\beta (T-{T}_{ref})$$

19 $${I}_{SC}\left(T\right)={I}_{SC,ref}+\alpha (T-{T}_{ref})$$

${V}_{OC}$: Open-circuit voltage at reference temperature, ${I}_{SC}$: Short-circuit current at reference temperature, $\beta$: Temperature coefficient of the open-circuit voltage.

### Wind turbine

The wind turbine system produces electric energy by driving the blades of a turbine, which rotate with an electrical generator. As the wind flies across those blades, they spin and voila—kinetic energy in the house. This mechanical energy is then delivered to the permanent magnet synchronous generator since it is chosen due to its cost-effectiveness, low maintenance, and small size^13,14^. The characteristics of wind turbine are shown in Fig. 3b.

The formula expressing the power harnessed from the wind can be articulated as follows:20

The converted power of the wind by the turbine which can be called as mechanical power and can be given as21where ${C}_{p}$ is the power coefficient which determines the amount of wind power converted into mechanical power and whose empherical formula can also be given a

22 $${C}_{p}=\frac{1}{2}(\gamma -0.022{\beta }^{2}-5.6){e}^{-0.17\gamma }$$

where $\beta$ is the pitch angle of the blade and $\gamma$ is tip speed ratio of the turbine, which can also be given as ratio of wind speed to the turbine angular speed and can be expressed as

23 $$\gamma =\frac{v}{w}$$

The swept area A is the area covered by the rotating blades and is given by

24 $$A=\pi {R}^{2}$$

The tip speed ratio λ is the ratio of the speed of the tip of the blade to the wind speed and is given by

25 $$\uplambda =\frac{wR}{\vartheta }$$

The relationship between angular velocity $w$ and power P can be used to find the mechanical power and torque

26 $$P=Tw$$

Air density ρ can change with altitude and temperature. It can be calculated using the ideal gas law

27 $$\uprho =\frac{P}{RT}$$

### Flywheel

A flywheel, a mechanical battery formed by mass rotating around an axis. Its uses include being a solution for power quality problems (e.g., fluctuation, interruption of wind energy systems), providing an option to give energy backup and having the ability to contribute in voltage sag management thanks for its fast reaction time. The use of flywheels is also common in uninterruptible power supply (UPS) applications. Among the most efficient and environmentally friendly energy storage systems researched, the flywheel battery subsystem possesses superior features such as high efficiency, long lifespan potential with low maintenance costs, storage uptime viability (energy), fast charge/discharge speed, time commitments, etc. Flywheel Energy Storage Systems, for instance. Interact with power electronic equipment, notably DC/AC bidirectional converters; that’s why they can easily combine into imperfect distributed systems due to the variety in operational conditions and this imperishes an interconnected system support phase-lock loop filter tuning method against unimpaired networks as per its local measurement and controls theory provisions. Working on a mechanical principle, flywheel stores kinetic energy in the form electrical energy^15^. The flywheel gives off the spinning mass (rotor) kinetic energy output and is then converted into electrical power via a bidirectional power converter. The energy storage part requires the rotor—essentially a flywheel in this application—to be spun up very quickly, and releasing that energy is done by decelerating the rotor until it stops turning altogether. The energy stored in the flywheel is directly proportional to rotor mass and angular velocity^16,17^.

This relationship is mathematically expressed by the equation for stored kinetic energy:

28 $$E=0.5J{W}^{2}$$

where *W* represents the rotor angular velocity and *J* denotes the moment of inertia, defined as:

29 $$J=Km{r}^{2}$$

where *m* represents the rotor mass (kg); *r* is the rotor radius; *K* is an inertial constant that depends on the rotor shape.

The angular velocity w is related to the rotational speed in revolutions per minute (RPM) by:

30 $$w=\frac{2\pi N}{60}$$

The power P output of a flywheel can be described by:

31 $$P=Tw$$

The time t it takes to discharge or charge the flywheel can be estimated by:

32 $$t=\frac{E}{P}$$

Energy losses in a flywheel system are primarily due to friction and air resistance. The efficiency η can be calculated by considering these losses:

33 $$\upeta =\frac{{E}_{out}}{{E}_{in}}\times 100\%$$

The rotational dynamics of a flywheel can be described by Newton’s second law for rotation.

34 $$T=I\alpha$$

To design a flywheel with a specific energy capacity, rearrange the kinetic energy equation.

35 $$m=\frac{2E}{{r}^{2}{w}^{2}}$$

These equations are essential for the design, analysis, and optimization of flywheel energy storage systems, ensuring they can effectively store and release energy as part of a hybrid MG system that includes photovoltaic arrays and wind turbines.

### Power flow management between the sources

To achieve stability, efficiency, and reliability, power flows between the various components of a wind turbine are controlled by a supervising controller based on control logic, for instance, fuzzy or predictive controllers.

#### Operation modes

Condition System Response

PV > Load Surplus goes to flywheel (store) or grid (export).

Wind > Load Any surplus goes into flywheel or grid.

PV + Wind < Load Flywheel releases to meet the vacuum.

PV/Wind Not Available Flywheel supports load (discharges).

#### Grid-connected mode

Excess sold to grid; shortfall purchased from grid.

#### Standalone mode

Need to match generation and storage moment by moment.

## MATLAB implementation of proposed MG system

PV-based MGs, the system is implemented using MATLAB by way of a model that simulates what happens when it is all together—photovoltaic (PV), micro-turbine, and flywheel; all these disparate component systems are accounting for their dynamics, figured out as possible interactions. In MATLAB, a modular representation can be created to model each subsystem that allows for examining influences on the behaviours of the individual powertrain and system performance. To design the PV system, MATLAB provides tools to model the photovoltaic array (including parameters such as solar irradiation level, temperature effects, and electrical characteristics of panels). Modelling of the micro-turbine module can be performed using suitable data models considering wind speed, turbine efficiency, and dynamic modelling for the generator. Finally, another feature of MATLAB is the ability to model energy storage systems, enabling us to use this tool for representing how the flywheel behaves in that MG. In MATLAB, you need to connect the several blocks that represent PV arrays, micro-turbines and flywheel storage in an integrated system model as well as implement the control strategies. A similitude strategy can’t accomplish this, yet MATLAB itself gives several functionalities intended for modelling state space matrices from linear algebra (using transfer functions) via specifying “subsystem” outputs regarding certain inputs concerning other subsystems, in addition to corresponding multiplicative/connection/matricial properties. This allows for a holistic examination of how well the high penetration, real-time renewable MG is able to utilise its available sources as well as deal with inherent variations and disturbances while ensuring stability in operation^18,19,20^.

### PID controller

**Fig. 4:**
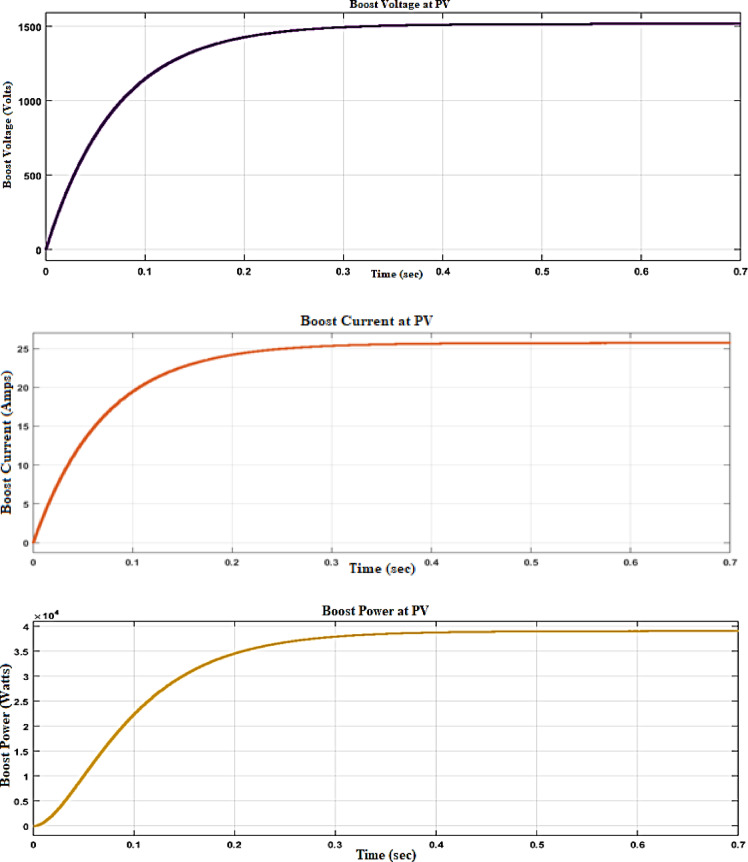
Voltage, current, and power of PV system with DC–DC converter with PID controller.

A Boost Converter with a PID (Proportional-Integral-Derivative) controller is designed in MATLAB using Simulink, a powerful tool for modelling, simulating, and analysing dynamic systems. The Boost Converter is a type of DC–DC converter that steps up (increases) the input voltage to a higher output voltage, making it suitable for applications where a higher voltage is required from a lower voltage source. In the Simulink model, the Boost Converter circuit consists of essential components such as an inductor, a diode, a capacitor, and a switch (typically a MOSFET). The input voltage is supplied to the inductor, and the switch controls the energy transfer to the load. The output voltage is controlled by adjusting the duty cycle of the switch, which is where the PID controller comes into play. The PID controller is implemented to regulate the output voltage by comparing the desired reference voltage with the actual output voltage. The controller adjusts the duty cycle of the switch in real-time to minimize the error between the reference and actual voltage. The PID controller parameters—Proportional (P), Integral (I), and Derivative (D) gains—are fine-tuned to achieve the desired response, ensuring stability, fast transient response, and minimal steady-state error. In the Simulink environment, the Boost Converter model is constructed using blocks representing the physical components and mathematical functions. The PID controller is designed using a PID Controller block, where the gains are set either manually or through an auto-tuning process. The entire system is then simulated to observe the dynamic response of the output voltage, demonstrating how effectively the PID controller maintains the desired output despite variations in load or input voltage, as shown in Fig. 4. This Simulink model serves as a valuable tool for understanding the behaviour of the Boost Converter and the impact of the PID controller on its performance, allowing for further optimization and testing under different conditions.

### Fuzzy controller

**Fig. 5:**
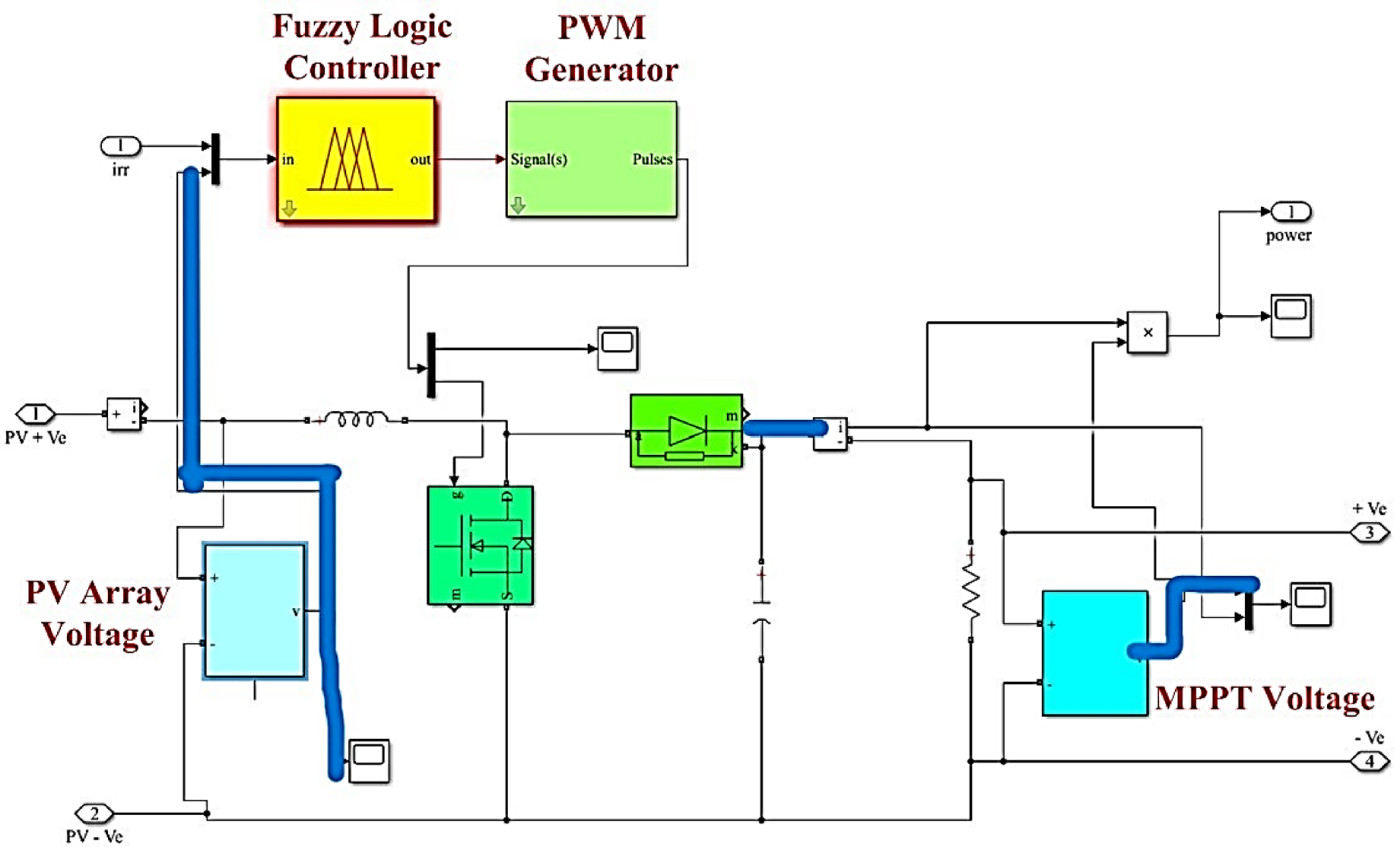
Proposed system with fuzzy controller.

The Simulink model, illustrated in Fig. 5, depicts a microgrid configuration featuring a PV power generation model. This model is intricately linked to a single-phase protection diode, enhancing the system’s resilience and safety measures. The combination of both alterable and constant radiation inputs advances and enriches the model’s refinement, allowing for an overall assessment of the MG’s performance under changing environmental conditions.

This layout not only warrants the studies of self-behaviour of the PV power generation models but also prospects their collaborative impact on the comprehensive system dynamics. The associated nature of these components within the Simulink model makes a smooth pathway for scrutiny of the MG’s feedback to changing PV radiation and magnifies our understanding of its usable characteristics^19,21^.

**Fig. 6:**
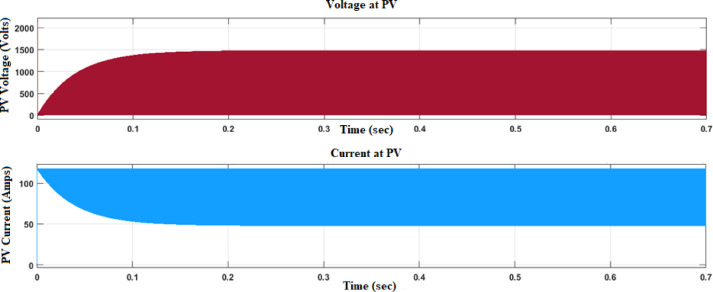
Voltage and current waveform at PV output with fuzzy controller.

**Fig. 7:**
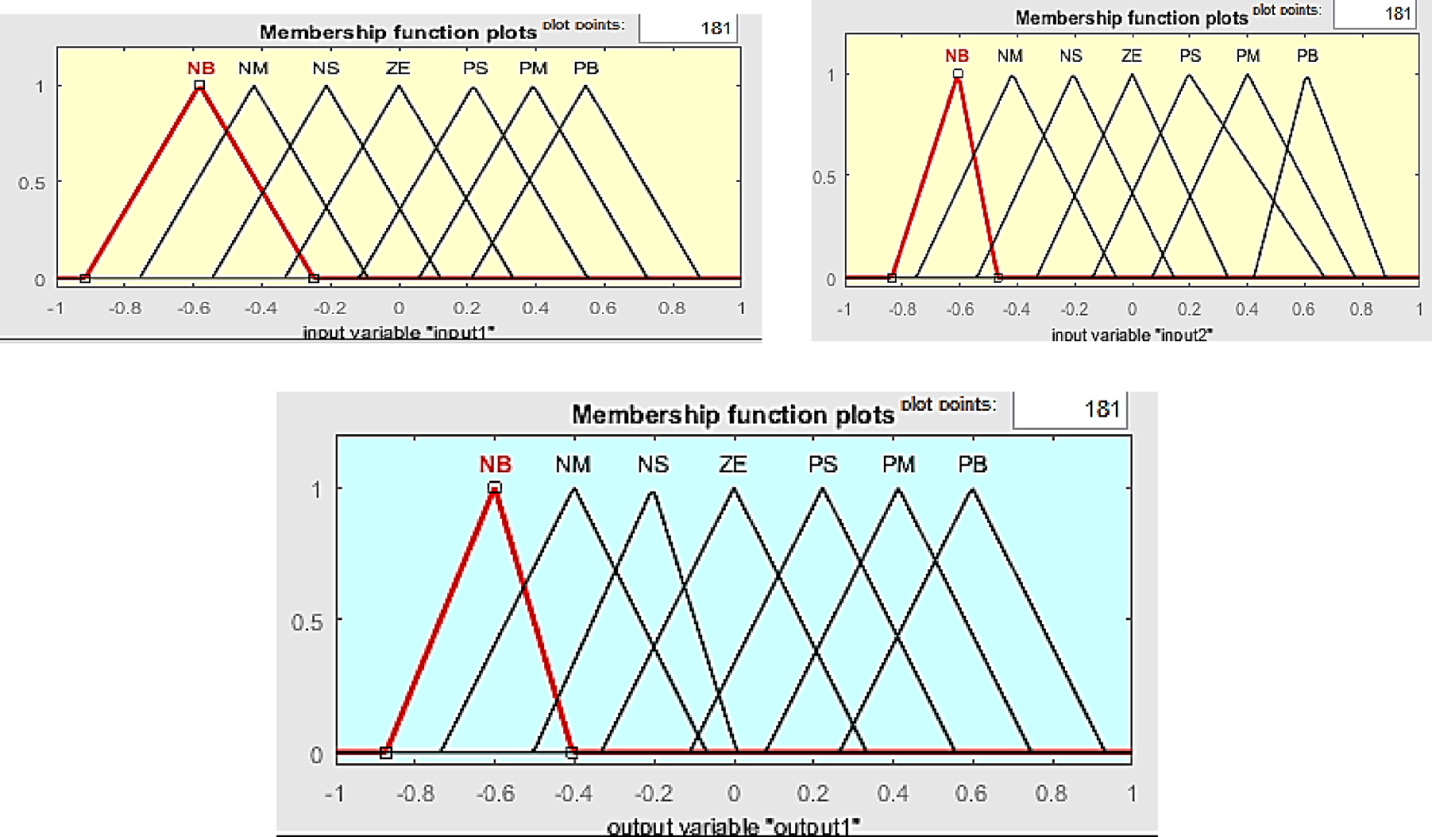
Fuzzy membership function plots.

Figure 6 demonstrates the voltage and current waveforms at the output of the (PV) system. The voltage waveform outlines the change in electrical potential over time, reviewing the alternating cycles of energy generation from the PV panels. Figure 7 shows the fuzzy membership functions applied to the system.

**Fig. 8:**
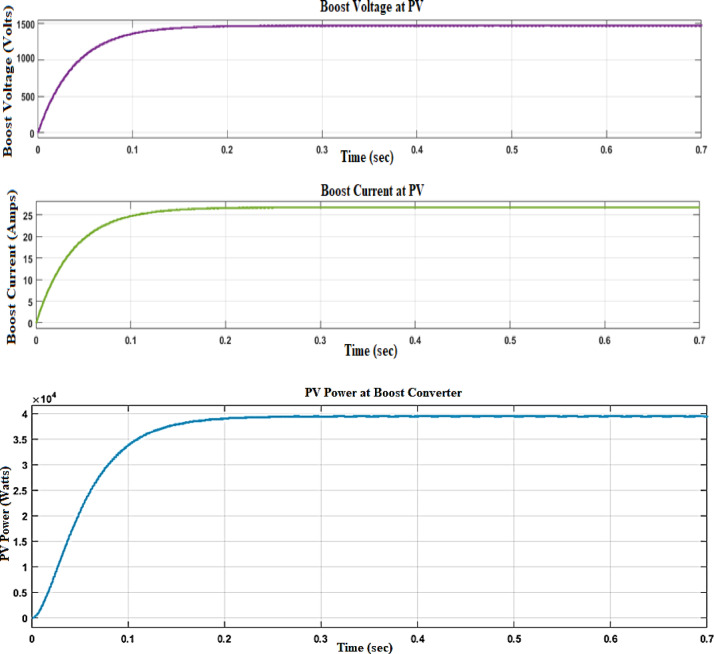
Voltage, current, and power waveform at the DC–DC side of PV with fuzzy controller.

**Fig. 9:**
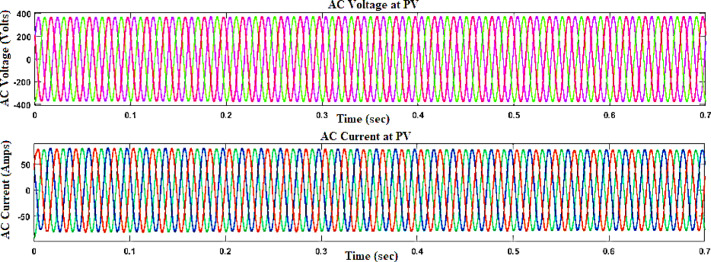
Voltage, current waveform at DC–AC side of PV with fuzzy controller.

Figure 8 depicts the voltage, current, and power waveforms at the DC–DC converter side of the (PV) system. The voltage waveform shows us the changes in electrical potential at the output side of the DC–DC converter over time. This waveform is important for forming an opinion on the stability of the voltage supply, making sure it meets the essential specifications for downstream components. Figure 9 depicts the voltage, current, and waveforms at the DC–AC converter side of the (PV) system.

**Fig. 10:**
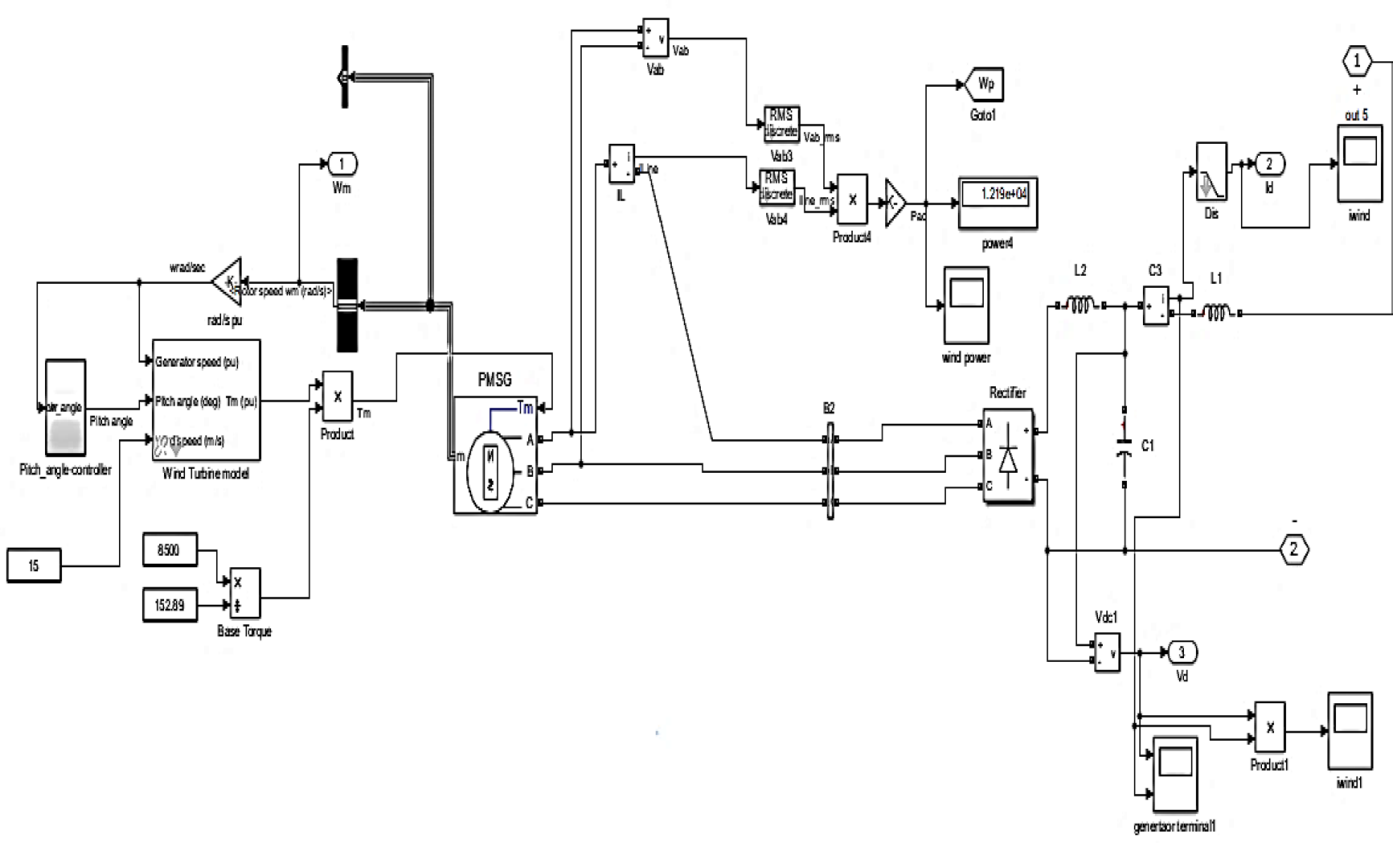
MATLAB model of a wind energy system with a DC–DC converter.

A collection of group variables defines a wind turbine model. Consequently, one of these symbols is the generator speed, which is represented as an input variable and measured in units. The operating principle of the wind turbine is based on its capacity to generate mechanical power by converting kinetic energy into mechanical force, as calculated throughout. In this manner, it becomes possible to emphasize the relationship between the wind turbine dynamic properties and the performance of the PMSG, and, as a result, the RES can be successfully interlinked with the system, as it is in Fig. 10^22,23^.

**Fig. 11:**
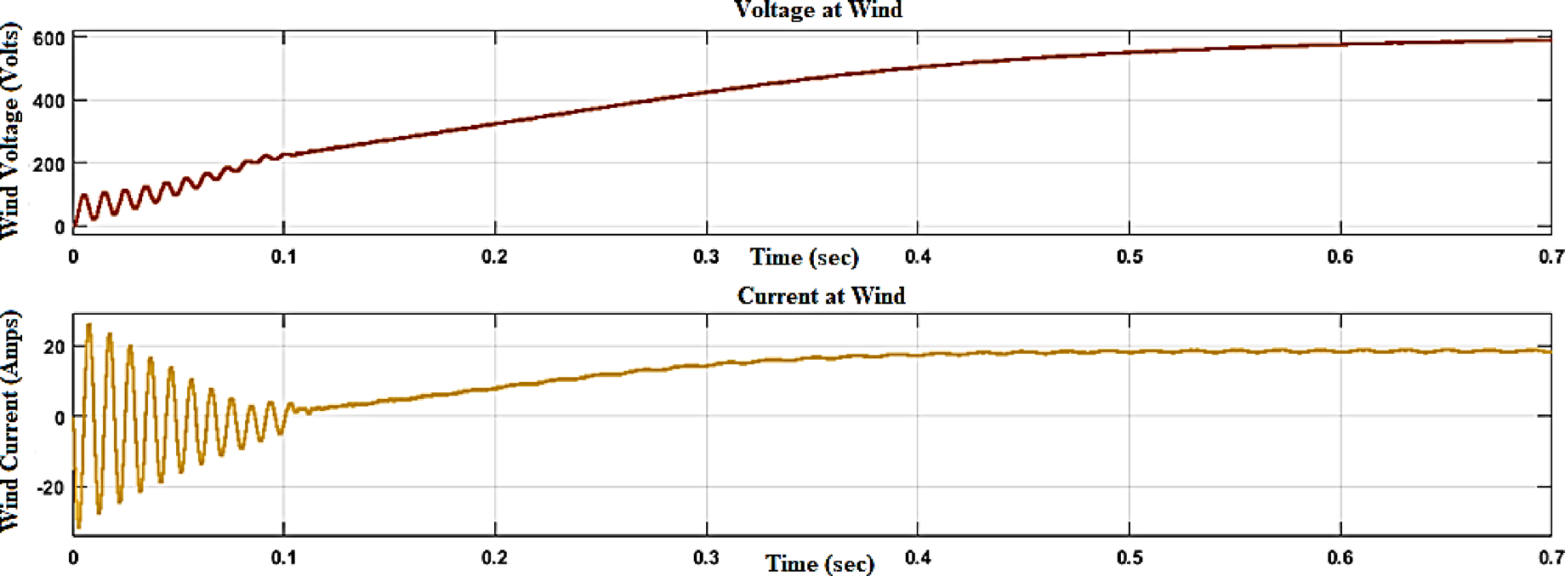
Voltage and current waveform at wind output.

In Fig. 11, the voltage and current waveforms at the wind turbine output are shown, providing a detailed look into the electrical characteristics of the wind energy conversion system. The voltage waveform describes the variation in electrical potential produced by the wind turbine over time, reflecting the intermittent nature of wind energy.

**Fig. 12:**
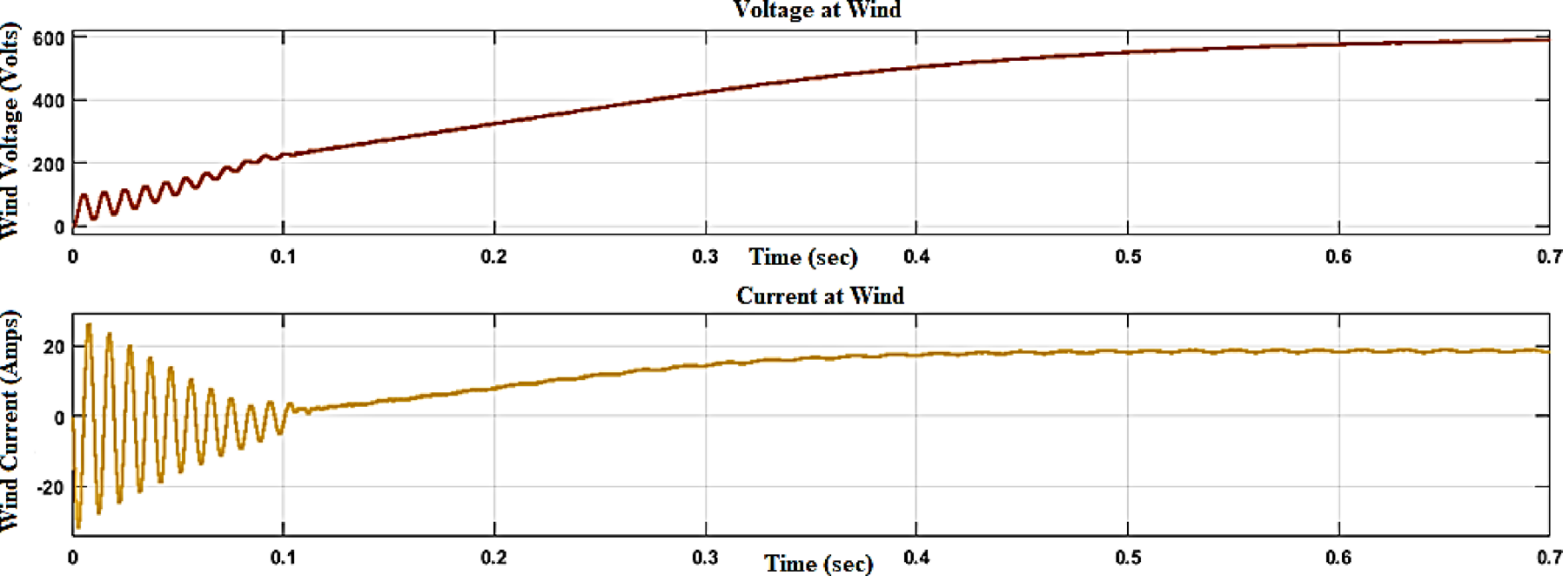
Voltage, current waveform at DC–DC side of wind system.

**Fig. 13:**
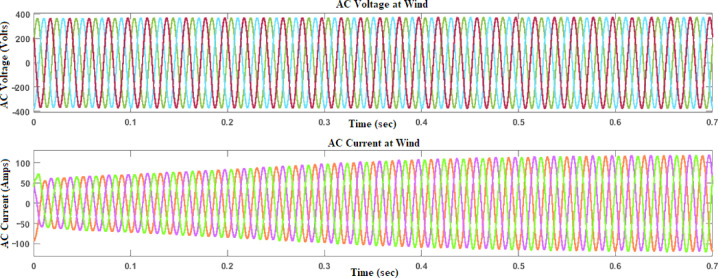
Voltage, current waveform at DC–AC side of wind system.

**Fig. 14:**
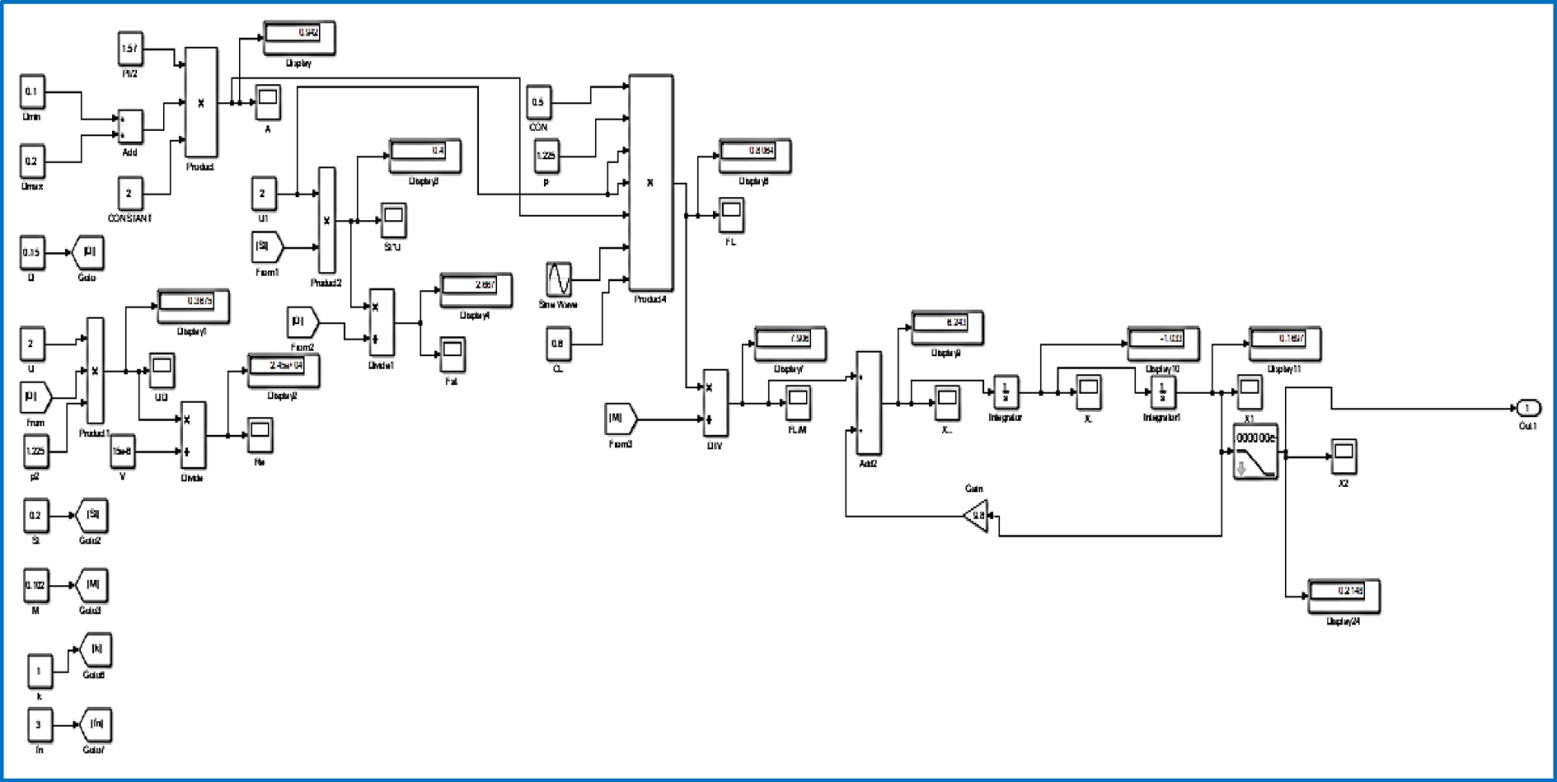
MATLAB model of the Flywheel energy system with DC–DC converter.

Figures 12 and 13 reflect the voltage and currents of the Wind system converter. Since MATLAB provides the users with tools and libraries for modelling mechanical systems, it is possible to describe the action of the flywheel in terms of converting its kinetic energy and subsequently storing it in the form of electrical power through a bidirectional power converter. Key parameters of such a system would be the moment of inertia of the flywheel and its corresponding speed of rotation, as shown in Fig. 14. In conclusion, the described approach would enable a detailed evaluation of the flywheel energy storage’s operation, providing valuable information for assessing the viability of such technology in minimizing energy fluctuations within the MG^23,24^.

**Fig. 15:**
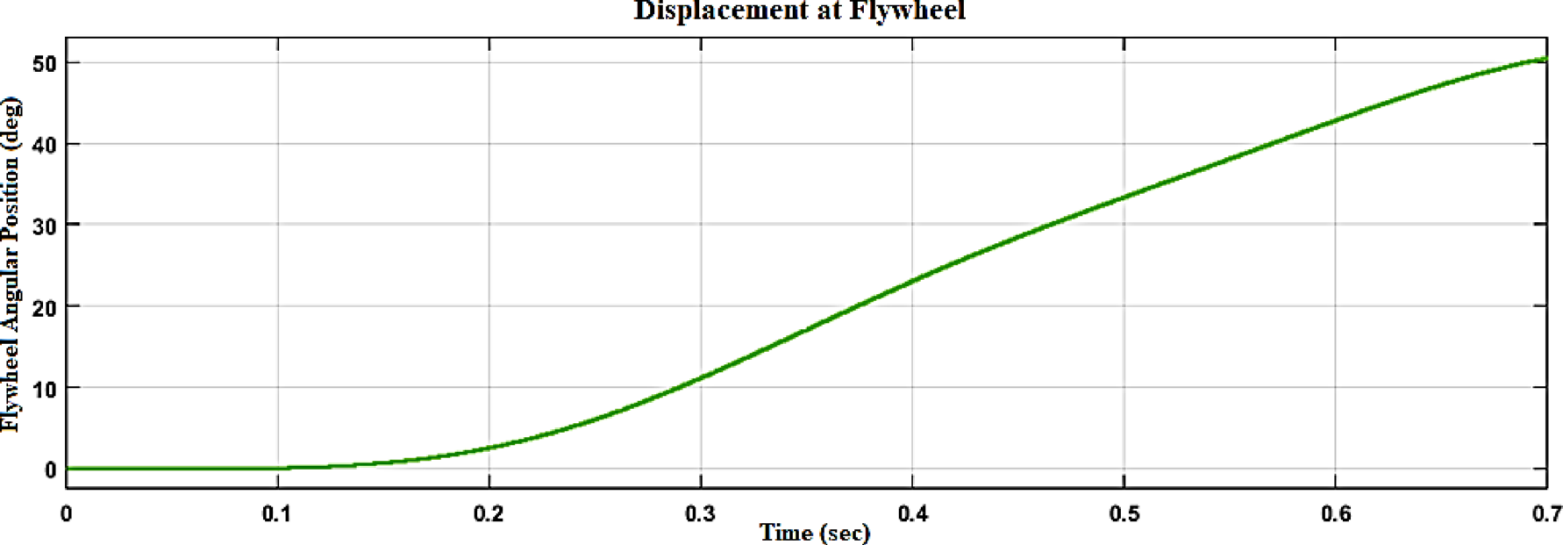
Displacement waveform at Flywheel.

**Fig. 16:**
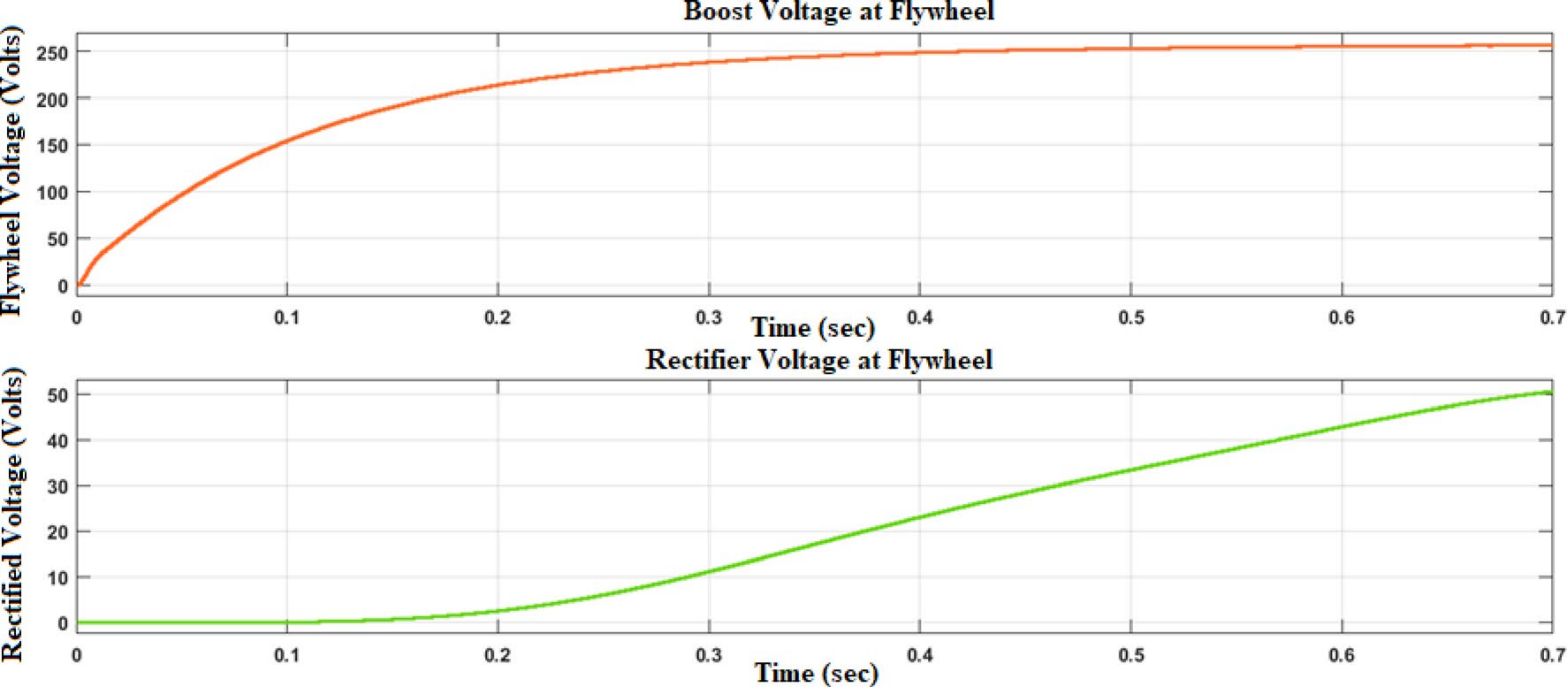
Voltage, current waveform at DC–DC side of flywheel system.

**Fig. 17:**
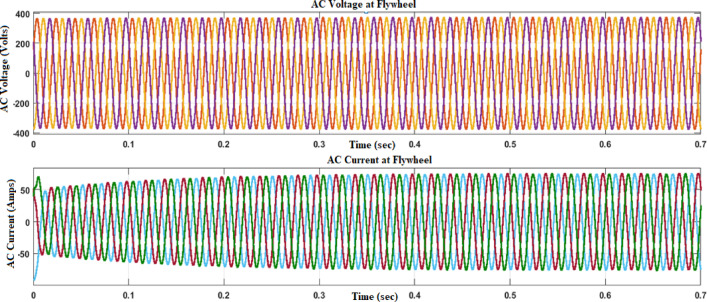
Voltage, current waveform at DC–AC side of flywheel system.

Figure 15 illustrates the displacement waveform at the flywheel, providing a detailed representation of the rotational movement of the flywheel over time. The displacement waveform showcases the variations in the flywheel’s angular position, reflecting the continuous spinning motion initiated by the stored kinetic energy. Figures 16 and 17 reflect the waveforms of the inverter side of the flywheel storage system^25,26^.

### Performance analysis and comparison

PID Controller-based system: As shown in Fig. 4, this system exhibits faster responses but is less stable, with higher oscillations and reduced voltage, current, and power, compared to the fuzzy system when renewable input changes suddenly.

Figure 4 depicts the voltage, current, and power waveforms at the DC–DC converter side of the (PV) system, the entire system is then simulated to observe the dynamic response of the output voltage, demonstrating how effectively the PID controller maintains the desired output despite variations in load or input voltage. The output voltage is controlled by adjusting the duty cycle of the switch, which is where the PID controller comes into play. The PID controller is implemented to regulate the output voltage by comparing the desired reference voltage with the actual output voltage. The controller adjusts the duty cycle of the switch in real-time to minimize the error between the reference and actual voltage. The PID controller parameters: Proportional (P), Integral (I), and Derivative (D) gains are fine-tuned to achieve the desired response, ensuring stability, fast transient response, and minimal steady-state error. In the Simulink environment, the Boost Converter model is constructed using blocks representing the physical components and mathematical functions. The PID controller is designed using a PID Controller block, where the gains are set either manually or through an auto-tuning process.

*Fuzzy Results* As shown in Fig. 7 smoother responses, reduced oscillations, better voltage, current and power under uncertainties are observed.

Figure 7 depicts the voltage, current, and power waveforms at the DC–DC converter side of the (PV) system. The voltage waveform shows us the changes in electrical potential at the output side of the DC–DC converter in course of time. This waveform is important for forming an opinion for the stability of the voltage supply, make sure it meets the essential specifications for downstream components.

The comparison of characteristic features of PID and Fuzzy is shown in Table 1.

**Table 1:** Comparative analysis of PID versus fuzzy.

Criteria	PID controller	Fuzzy controller
System stability	Good for linear, stable conditions; performance degrades under nonlinearity	Excellent; adapts well to nonlinear & uncertain conditions
Response time	Faster in simple cases; overshoot may occur under disturbances	Slightly slower, but smoother transition with less overshoot
Voltage/frequency Regulation	Works well if tuned, but tuning is difficult under varying inputs	More robust against varying inputs (solar irradiance, wind speed)
Robustness	Sensitive to parameter changes	Highly robust to parameter variations and load changes
Implementation	Easy, low cost, less computational demand	Requires expertise, higher computational demand
Power quality (THD, harmonics)	Higher fluctuations possible	Lower fluctuations, better power quality

The performance comparison between a DC–DC converter fed with a PID (Proportional-Integral-Derivative) controller and a Fuzzy controller, as presented in Table 2, highlights the differences in key operational parameters such as voltage, current, power, settling time, rise time, and ripple percentage. The output voltage with the PID controller is recorded at 1520 V, while the Fuzzy controller achieves a slightly higher output of 1570 V. Similarly, the current is 25.6A with the PID controller and increases to 26.9A with the Fuzzy controller. This indicates that the Fuzzy controller is more effective in boosting both voltage and current, thereby enhancing the converter’s performance. The power output, calculated as the product of voltage and current, shows a notable difference between the two controllers. The PID controller delivers 3.9 × 10^4^ W (or 39,000 W), whereas the Fuzzy controller provides a higher output of 4.1 × 10^4^ W (or 41,000 W). This suggests that the Fuzzy controller can extract and deliver more power from the converter, making it more efficient in terms of energy conversion^27^. When analysing the dynamic response, the Fuzzy controller outperforms the PID controller in both settling time and rise time. The settling time, which is the time taken for the output to stabilize within a certain percentage of its final value, is shorter with the Fuzzy controller at 0.22 s compared to 0.35 s with the PID controller. Similarly, the rise time, which measures how quickly the output reaches its final value from a specified initial value, is faster with the Fuzzy controller at 0.19 s versus 0.31 s for the PID controller. These results demonstrate that the Fuzzy controller provides a quicker and more responsive control, leading to faster system stabilization. Ripple, representing the variations in the output voltage and current, is another crucial performance metric. The PID controller exhibits a ripple percentage of 7%, whereas the Fuzzy controller reduces this to 3%. Lower ripple is desirable as it indicates a smoother and more stable output, which is particularly important in sensitive electronic applications. The Fuzzy controller’s ability to minimize ripple further underscores its superiority in ensuring a stable and consistent production.

**Table 2:** Performance comparison DC–DC converter fed with PID and fuzzy controllers.

Parameter	PID controller	Fuzzy controller
Voltage (V)	1520 V	1570 V
Current (A)	25.6A	26.9A
Power (W)	3.9*104W	4.1*104W
Settling time (s)	0.35 s	0.22 s
Rise time (s)	0.31 s	0.19 s
% Ripples	7%	3%

## Conclusion

The design and interconnection of an MG system with integrated solar (PV) technologies, palm-sized turbines, and a flywheel is a viable solution to decentralize power generation. Since each of these components tackles significant challenges in power quality, energy backup, and voltage control, the combination is synergistic. PV panels provide a source of clean and renewable power, the system can draw energy from wind as well, with the help of micro turbines, therefore increasing its own flexibility. The flywheels provide a good storage energy element that enables the proper control of inconsistent power sources and efficiency to balance direct current. Implementation The MATLAB-implemented MG concept offers a promising path for meeting energy requirements, enhancing grid resilience, and helping secure a sustainable energy future. Future research on these grid-edge MG systems is necessary to validate the theoretical findings as well as enhance their feasibility in practice. The comparison highlights the advantages of using a Fuzzy controller over a PID controller in a DC–DC converter. The Fuzzy controller not only achieves higher voltage, current, and power but also offers faster dynamic response and significantly reduces output ripple. These benefits make the Fuzzy controller a more effective and efficient choice for optimizing the performance of DC–DC converters, particularly in applications requiring precise and rapid control.

## Data Availability

The datasets generated and/or analysed during the current study are available from the first author on request.

## List of symbols

Ppv: Output power of the PV array (watts)
A: Total area of the panel (active area exposed to sun) (m^2^)
G: Solar irradiance (W/m^2^)
ηPV: Efficiency of the solar panel (%)
I: Output current of the solar cell (Amperes)
Iph: Photogenerated current (Amperes)
I0: Reverse saturation current of the diode (Amperes)
V: Output voltage across the solar module (Volts)
Rs: Series resistance (Ohms)
Rsh: Shunt resistance (Ohms)
Q: Elementary charge of an electron (1.602 × 10^−^^19^)
K: Boltzmann constant (1.381 × 10^−^^23^)
T: Cell temperature (Kelvin)
Cp: Power coefficient
ƿ: Air density (kg/m^3^)
A: Swept area of the wind turbine (m^2^)
V: Wind speed at the hub height (m/s)
Eflywheel: Kinetic energy stored in the flywheel (Joules)
J: Moment of inertia (kg-m^2^)
W: Angular velocity of the rotating flywheel (rad/s)
M: Mass of the flywheel (Kg)
R: Radius of the flywheel (Meters)
∝: Temperature coefficient of the short-circuit current (%/°C)
Voc: Open-circuit voltage of a solar cell (Volts)
Isc: Short-circuit current at reference temperature (A/mA)
β: Temperature coefficient of the open-circuit voltage (mV/°C)
